# Commencements

**Published:** 1894-07

**Authors:** 


### COMMENCEMENTS.

### National University—Dental Department.

The annual commencement exercises of the Dental Department of the National University were held, in connection with those of the Medical Department, at Metzerott's Music Hall, Washington, D. C., on Tuesday evening, May 15, 1894.

The address to the graduating classes was delivered by Prof. George C. Ober, M.D., and the valedictory address by J. Edwin Waterbury, D.D.S.

The number of matriculates for the session was twenty-eight.

The degree of D.D.S. was conferred on the following graduates by the Chancellor of the University, Hon. Arthur MacArthur, LL.D.:

| NAME. | RESIDENCE. | NAME. | RESIDENCE. |
|---|---|---|---|
| Barney Babcock | New York | John A. McAfee | Texas |
| Ransom L. Caton | Alabama | John B. Reeves | Illinois |
| William C. Cannon | Illinois | Walter F. Schricker | Missouri |
| Frederick F. Daly | Dist. of Columbia | J. Edwin Waterbury | California |

Total, 8.

### Southern Medical College—Dental Department.

The seventh annual commencement exercises of the Dental Department of the Southern Medical College were held in College Hall, Atlanta, Ga., February 27, 1894.

The annual address to the students was delivered by T. W. Latham, and the valedictory by W. C. Mattox.

The number of matriculates for the session was thirty-one.

The degree of D.D.S. was conferred on the following graduates by T. S. Powell, M.D., President of the Board of Trustees:

| NAME. | RESIDENCE. | NAME. | RESIDENCE. |
|---|---|---|---|
| W. B. Fuller | Florida | W. C. Mattox | Tennessee |
| Arno Knorr | Georgia | W. H. Wooldridge | Texas |

Total, 4.

### Birmingham Dental College.

The commencement exercises of the Birmingham Dental College were held in Seal's Music Hall, Birmingham, Ala., Wednesday evening, March 7, 1894.

The address to the graduating class was delivered by Capt. W. C. Ward, and the valedictory by D. C. Cosby, D.D.S.

The number of matriculates for the session was twenty-seven.

The degree of D.D.S. was conferred on the following graduates by the Dean, T. M. Allen, D.D.S.:

| NAME. | RESIDENCE. | NAME. | RESIDENCE. |
|---|---|---|---|
| Drury C. Cosby | Alabama | William P. Stinson | Alabama |
| John H. Rice | Alabama | | |

Total, 3.

### University of Pennsylvania—Department of Dentistry.

The fifteenth annual commencement of the Department of Dentistry of the University of Pennsylvania was held in the American Academy of Music, Philadelphia, on Thursday, June 7, 1894.

The number of matriculates for the session was two hundred and thirty-one.

An address was delivered by Louis A. Duhring, M.D., Professor of Skin Diseases.

The degree of D.D.S. was conferred on the following graduates by William Pepper, M.D., LL.D., Provost of the University:

| NAME. | RESIDENCE. |
|---|---|
| Antonio d' Avila | Brazil |
| Miles C. Barner | Pennsylvania |
| Marcel Barral | France |
| John R. A Berry | Switzerland |
| George R. Blanchard, jr. | Pennsylvania |
| Hans Block | Germany |
| Frederick L. Bogue | New Jersey |
| Frederic H. Bowers | Illinois |
| D. Paton Boyd, L.D.S | Scotland |
| Harry B. Butler | New York |
| John J. Cush | Pennsylvania |
| F. A. Delabarre, A.B. | Massachusetts |
| Wilson D. DeLong, M.D. | Pennsylvania |
| John Dunlop, L.D.S | Scotland |
| Theodore C. Edgar | Iowa |
| Rudolph Erler | New York |
| George H. Finch | Virginia |
| Harley B. Fowler | Wisconsin |
| Martin Freund, Ph.D | Germany |
| Mendel Friedland | Russia |
| Lewis H. Gilman | Pennsylvania |
| August Hafner | Switzerland |
| Wilhelm Harres | Germany |
| J. Irvin Hatch | Pennsylvania |
| E. Foster Hazell | Illinois |
| Joseph M. Hill | Pennsylvania |
| John E. Horgan | Pennsylvania |
| Raymond W. Hunter | Massachusetts |
| Arthur T. Kenney | Australia |
| Emil W. Kuehn | Minnesota |
| Robert Lavanchy | Switzerland |
| Frederick A. Lewis | Iowa |
| Marquis D. Littig | Iowa |
| J. T. Lippincott, D.D.S. | Pennsylvania |
| George E. Longeway | Canada |
| Harry G. Maize | Pennsylvania |
| Edward J. Marfing | Pennsylvania |
| Walter S. Marlow | England |
| Herant B. Matteossian, A.B. | Turkey |
| Carl Mosberg | Germany |
| Albert Newgarden | Pennsylvania |
| Peter O'Donnell | Pennsylvania |
| John P. Oliver, L.D.S. | England |
| Thurlow G. Patterson | New York |
| Charles F. Portz | Pennsylvania |
| J. Martin Raugh, D.D.S | Iowa |
| Guy C. Robb | Pennsylvania |
| John A. Robeson | North Carolina |
| Walther Rosendahl | Germany |
| Ernst Rosenthal | Germany |
| Philip H. Seibel, M.D | California |
| Lourival M. de Souza | Brazil |
| D. Ambrose Stein | Pennsylvania |
| Charles E. Stine | Pennsylvania |
| John W. Storer | West Virginia |
| Campbell C. Taggart | Pennsylvania |
| Howard S. Taylor | Delaware |
| Fred. M. Thiesen | Germany |
| Harold Tyndale | Pennsylvania |
| Victor de Trey | Switzerland |
| Arthur D. Weakly | Illinois |
| B. Frank Witmer | Pennsylvania |
| William J. Wray | Pennsylvania |
| Wilson Zerfing | Pennsylvania |

Total, 64.

JUNE 16, 1893.

| NAME. | RESIDENCE. |
|---|---|
| Joseph M. McDowell | Pennsylvania |

Total, 65.

### College of Dental Surgery of the University of Michigan.

The nineteenth annual commencement of the Dental Department of the University of Michigan was held, in connection with the commencement exercises of the other departments, in University Hall, Ann Arbor, Thursday, June 28th, 1894.

The number of matriculates for the session was one hundred and eighty-five.

The annual commencement oration was delivered by George Herbert Palmer, A.M.

The degree of D.D.S. was conferred on the following candidates by the President of the University, James B. Angell, LL.D.:

| NAME. | RESIDENCE. |
|---|---|
| Delia C. O. Adams | Michigan |
| Frank P. Adams | Indiana |
| Chas. Francis Amsden | Ohio |
| Otto Anderson | Michigan |
| Adelbert H. Babcock | Michigan |
| Edwin Irving Backus | Indiana |
| Andrew S. Bailey, B.S., Lawrence University | Wisconsin |
| Roy Edwin Bailey | Michigan |
| Fred. Wm. Blake | Michigan |
| Henry M. Bridgman, | Natal, Africa |
| Damon Isaiah Butler | Michigan |
| Thomas S. Buzzard | Michigan |
| Anthony J. Casey | Michigan |
| Chas. Douglas Cassidy | New York |
| Frederick H. Codding | Michigan |
| Estus Hammond Coller | Michigan |
| Gerald Willard Collins | Michigan |
| Robt. Edgar Davies | New York |
| Frank B. Dawley | Michigan |
| James King Douglas | Ohio |
| William Booth Elster | Nebraska |
| Edward Leigh Gedney | Minnesota |
| William E. Goucher | Michigan |
| Myron Perry Green | Michigan |
| Harry Loyal Griswold | Illinois |
| Alfred Whipple Hall | Michigan |
| William Anthony Hart | Michigan |
| Garrett S. Hartley | Pennsylvania |
| Charles P. Haselden | Massachusetts |
| George Elba Hathaway | Michigan |
| William J. Higgins | Michigan |
| John Louis Hoover | Indiana |
| Homer F. Hussey, Ph.B., Earlham College | Indiana |
| George Renshaw Johnson | Michigan |
| George W. Kenson | Wisconsin |
| Allen Huyler Kessler | Michigan |
| Joseph Lathrop, jr. | Michigan |
| Chas. Cummings Lick | Michigan |
| Mary Linde | Germany |
| Robt. Bruce Mackenzie | Michigan |
| Michael J. McCormick, Rockburn | Province of Quebec |
| Chas. A. McGettigan, jr | California |
| James Archibald McIndoe | Wisconsin |
| Walter Chas. McKinney | Michigan |
| Anna Katharine Miller | Michigan |
| Chas. Lester Mitchell | Ohio |
| Albert Francis Monroe | Michigan |
| Geo. McWilliams Moore | Wisconsin |
| Miles Jacob Moyer | Michigan |
| Allen Eugene Mulder | Michigan |
| Forest Joseph Overholt | Michigan |
| Barnum Herbert Pearce | Ohio |
| Benj. Franklin Pearce | California |
| Geo. Andrew Servis | Michigan |
| Carrie M. Stewart | Texas |
| Harvey A. Sturdevant | Colorado |
| Dean Nathaniel Swift | Ontario |
| Chas. Henry Terry | Illinois |
| Chas. Reed Vanderbelt | New York |
| Albert Wesley Weible | North Dakota |
| Chas. Traver Whinery | Ohio |
| Walter Morey Wilkins | Michigan |
| Wallace V. Wolvin | Michigan |
| George Philip Wurster | Michigan |

Total, 64.

### Columbian University—Dental Department.

The seventh annual commencement of the Dental Department of the Columbian University was held at University Hall, Washington, D. C., on Thursday, May 3, 1894, at 8:30 P. M.

The address to the graduates was delivered by Prof. R. B. Donaldson, D.D.S., and the valedictory by John H. Galloway, D.D S.

The number of matriculates for the session was twenty-eight.

The degree of D.D.S. was conferred on the following graduates by J. C. Welling, LL.D., President of the University:

| NAME. | RESIDENCE. |
|---|---|
| Chester H. Beatty | Dis't of Columbia |
| John H. Galloway | Iowa |
| Walter A. Low | Virginia |
| William A. Lyon | Connecticut |
| Herbert MacNamee | New York |
| Frederick W. Parker | New York |
| Charles S. Rice, M.D | Pennsylvania |
| T. Guy Songster | Dis't of Columbia |

Total, 8.

